# Understanding the Role of Dysfunctional and Healthy Mitochondria in Stroke Pathology and Its Treatment

**DOI:** 10.3390/ijms19072127

**Published:** 2018-07-21

**Authors:** Hung Nguyen, Sydney Zarriello, Mira Rajani, Julian Tuazon, Eleonora Napoli, Cesar V. Borlongan

**Affiliations:** 1 College of Medicine, University of South Florida Morsani, Tampa, FL 33612, USA; hungvuthienn@health.usf.edu (H.N.); zarriello@health.usf.edu (S.Z.); mira.rajani@emory.edu (M.R.); jptuazon@health.usf.edu (J.T.); 2 Department of Molecular Biosciences, School of Veterinary Medicine, University of California, Davis, CA 95616, USA

**Keywords:** cerebral ischemia, blood brain barrier, endothelial cells, impaired mitochondria, neurovascular unit, regenerative medicine, stem cell therapy, transfer of healthy mitochondria, vasculature

## Abstract

Stroke remains a major cause of death and disability in the United States and around the world. Solid safety and efficacy profiles of novel stroke therapeutics have been generated in the laboratory, but most failed in clinical trials. Investigations into the pathology and treatment of the disease remain a key research endeavor in advancing scientific understanding and clinical applications. In particular, cell-based regenerative medicine, specifically stem cell transplantation, may hold promise as a stroke therapy, because grafted cells and their components may recapitulate the growth and function of the neurovascular unit, which arguably represents the alpha and omega of stroke brain pathology and recovery. Recent evidence has implicated mitochondria, organelles with a central role in energy metabolism and stress response, in stroke progression. Recognizing that stem cells offer a source of healthy mitochondria—one that is potentially transferrable into ischemic cells—may provide a new therapeutic tool. To this end, deciphering cellular and molecular processes underlying dysfunctional mitochondria may reveal innovative strategies for stroke therapy. Here, we review recent studies capturing the intimate participation of mitochondrial impairment in stroke pathology, and showcase promising methods of healthy mitochondria transfer into ischemic cells to critically evaluate the potential of mitochondria-based stem cell therapy for stroke patients.

## 1. Therapeutic Options for Stroke

Currently, treatments for ischemic stroke are limited to eliminating occlusions and restoring blood flow via intravenous administration of thrombolytics like alteplase, a recombinant tissue plasminogen activator, in which effective application is restricted to a 4.5-h window [1]. An endovascular thrombectomy may supplement intravenous thrombolysis or serve as an alternative procedure to eradicate blockages if intravenous thrombolysis cannot be performed [1]. Negative outcomes of stroke can be mitigated by managing blood sugar, body temperature, and blood pressure [1]. However, not all stroke patients receive appropriate medical intervention in time, and certain individuals may not be eligible for thrombolysis [1,2]. Thus, it is imperative to develop additional therapeutic options for stroke [2]. Several small-molecule compounds have shown promising potential for treating stroke. Stachybotrys microspora triprenyl phenol-7 (SMTP-7), a plasminogen activator that exerts thrombolytic effects, produced abated infarct area, hemorrhages, and neurologic deficits in non-human primate stroke models [2,3]. Additionally, in a mouse stroke model, the small molecule NSI-189 increased neurogenesis, cell proliferation, neurotrophic factors, and behavioral recuperation, with the added benefit of having a six-hour time window for delivery after stroke [2,4]. Indeed, these small molecules may have the potential for developing more efficient stroke therapies to augment the meager arsenal of present stroke treatments.

## 2. Mitochondria and Stroke

Decades of biochemical studies have forged for mitochondria the definition of ‘energy powerhouse of the cell’, due to their critical role in the production of adenosine triphosphate (ATP), the principal molecule for the storage and transfer of energy in cells. However, being an integral part of multiple cellular signaling pathways, mitochondria have an equally critical role in energy metabolism regulation, cell cycle, survival and death, apoptosis, generation of reactive oxygen species (ROS), and calcium homeostasis [5,6]. The coupling of upstream oxidative metabolism (glycolysis, fatty acid beta oxidation, TCA cycle turnover) to oxidative phosphorylation (OXPHOS) generates approximately 90% of the total cellular energy demand [7,8].

Under physiological conditions during aerobic respiration, the leak of about 2% of the total electrons flowing across the ETC, prevalently from complexes I and III, leads to the generation of superoxide [9,10]. Superoxide and other reactive oxygen species (hydrogen peroxide, hydroxyl radical and derivatives) target and damage macromolecules like lipids, nucleic acids, and proteins, potentially contributing to the onset and progression of a number of diseases, like myocardial infarction, inflammatory conditions, certain cancers, atherosclerosis, as well as the physiological process of aging. 

The human central nervous system has an extremely high-energy demand (approximately 20% of the body’s total metabolic expenditure). The majority of this energy is spent on the principal neuronal function of firing action potentials, and neuronal communication through chemical synapses [11]. Accordingly, mitochondrial pathobiology might contribute to neurodegeneration in Alzheimer’s, Parkinson’s and Huntington’s disease [12,13], major psychiatric illnesses, including depression [14], schizophrenia [15], as well as neurodevelopmental disorders like autism spectrum disorder (ASD) [16,17]. Moreover, mitochondrial dysfunction, via diminished oxidative phosphorylation and energy production, may lead to the pathogenesis of monogenic genetic diseases like Anderson-Fabry disease, a disease which may generate an ischemic stroke [18,19,20].

Ischemic stroke is caused by thrombotic or embolic occlusion of a cerebral artery, resulting in the sudden loss of blood circulation to an area of the brain, with consequent loss of neurologic function. Ischemic stroke that is not treated promptly can cause necrosis of brain tissue, ultimately leading to disability and death [21,22]. Although aging increases the risk of stroke [23], stroke rates are also climbing in young adults, which comprise 10–15% of stroke patients. Stroke in young adults is especially concerning, as young people are often left disabled during their productive years [24]. Thrombolytics have been successful if administered within the 4.5-h treatment window. However, restoring the brain to pre-stroke conditions is challenging [25,26]. If thrombolytic treatment is not possible, a thrombectomy can be performed, although other alternative therapies are scarce [27].

Based on the critical role of mitochondria in neurons, and due to their susceptibility to brain ischemia/reperfusion injury, as well as their involvement in the cell death cascade [28,29], this review explores the contribution of mitochondria to the pathophysiology of stroke, and discusses the potential of mitochondria-based regenerative medicine for stroke therapy. 

## 3. Mitochondria, ETC, and OXPHOS

Mitochondria are essential to the life of cells due to their main role in energy production. The complexes that compose the ETC serve as the major structural and functional units of the mitochondrion, being sites of redox reactions that facilitate the phosphorylation of ADP to ATP [30]. As such, defects in ETC and OXPHOS can result in fatal consequences for the cell. Production of ROS, and consequent oxidative stress, is considered one of the major causes of degenerative processes. Through the formation of superoxide anion, hydrogen peroxide, and hydroxyl radicals [31], the mitochondria are major contributors of overall cellular ROS production [32], acting as key mediators of disease states [33], leading to cell damage and homeostatic disruption. 

The OXPHOS machinery consists of five large multi-subunit complexes (CI-CV), located in the heavily folded inner mitochondrial membrane and arranged into supercomplexes [34,35]. Of the ~90 subunits constituting the OXPHOS machinery, 13 are encoded by the maternally inherited mitochondrial DNA (mtDNA), while the rest are of nuclear DNA origin. Electron transfer from FADH_2_ and NADH to molecular oxygen ensues in the translocation of protons across the inner mitochondrial membrane at CI, CIII and CIV sites, giving rise to the electrochemical gradient sustaining ATP synthesis, ion translocation and protein import.

In humans. OXPHOS deficits account for about 1/5–10 000 births [36], and the individual complexes of the ETC play critical roles in the onset and progression of a number of pathological states. Although a thorough discussion of the existing mitochondrial disorders is outside of the scope of this review, it is worth mentioning that many of these diseases are characterized by damages at a neuronal level, with features like encephalopathy (Co-Enzyme Q10 deficiency, Complex I-IV deficiencies, Leigh disease, MIRA), epilepsy, seizures and ataxia (MERRF, MIRAS, Leigh disease, Friedreich’s ataxia), and stroke-like episodes (MELAS). 

Complex I (NADH dehydrogenase) has been implicated in a number of neurodegenerative disorders [37,38], with Complex I deficiencies being the most frequent defects ascribed to mitochondrial energy metabolism [39]. With its flavin (FMN)- and iron-sulfur clusters- moieties hosting subunits, Complex I is the major entry-point of electrons from NADH into the OXPHOS system via ubiquinone. During this process, the leakage of electrons and their premature transfer to oxygen may occur, making Complex I a critical site of superoxide production [40], leading to increased oxidative stress. In a vicious cycle, oxidative stress in turn leads to protein damage and compromises membrane integrity, affecting the maintenance of the mitochondrial membrane potential [41], resulting in mitochondrial depolarization, further precipitating the initial mitochondrial deficit.

Similarly, Complex II (succinate dehydrogenase) deficiencies set the stage for a range of clinical conditions, spanning from cancer, Leigh syndrome, cardiomyopathies, and infantile leukodystrophies [42]. Although deficits of succinate dehydrogenase are quite rare *per se*, accounting for around 2% of all respiratory chain defects, a critical role for Complex II has been established in the mediation of the induction of apoptosis associated with a defective ETC. Acidification caused by apoptosis-favoring compounds, such as the Fas ligand, is detected by Complex II, resulting in ROS production and cell death [43]. 

Complex III dysfunction has similar detrimental effects on the cell due to its critical role in establishing the proton motive force that is required for ATP synthase action. Inhibition or destruction of Complex III has been associated with pesticide exposure, causing a backup of electrons in the ETC and subsequent ROS production, leading to mitochondrion-mediated apoptotic cell death [44]. Of note, epidemiological studies have connected such pesticide exposure to Parkinsonian phenotypes [45]. Mutations in genes encoding cytochrome *b* or other subunits of Complex III have also been implicated in additional conditions such as exercise intolerance and ischemic cardiomyopathy [46,47].

Complex IV (cytochrome *c* oxidase), the ETC terminal enzyme, is responsible for reducing oxygen though the transfer of electrons from reduced cytochrome *c* [48]. Deficiencies in Complex IV make up a significant portion of respiratory chain defects [49]. Although mutations of the mtDNA coding for cytochrome *c* oxidase subunits are uncommon, Complex IV deficiencies inherited through autosomal recessive transmission appear more frequently, and are associated with phenotypes such as Leigh Syndrome, hypertrophic cardiomyopathy and myopathy, and fatal infantile lactic acidosis [48]. In addition, in instances of iron-deficiency, as seen in anemia, the loss of cytochrome *c* oxidase activity may ensue, aggravating the consequences of oxidative stress [50].

ATP synthase (commonly known as Complex V) plays a crucial role in mitochondrial function and morphology. The primary function of ATP synthase is synthesizing ATP from ADP using the proton electrochemical gradient. ATP synthase is also implicated in the maintenance of the mitochondrial cristae and in the formation of the permeability transition pore complex [51,52]. While Complex V defects are considered rare, they are generally extremely severe [53]. Qualitative and quantitative deficiencies characterize ATP synthase; the former involves structural modifications of the enzyme (e.g., imperfect assembly), the latter its levels [54,55]. Qualitative deficiencies are the result of mutations in mtDNA-encoded ATP synthase subunits causing the enzyme to either improperly assemble and/or function. These deficiencies manifest in many disorders such as neuropathy, ataxia, and retinitis pigmentosa (NARP), maternally inherited Leigh syndrome (MILS), and encephalo(cardio)myopathy [55,56]. Conversely, quantitative deficiencies occur in the presence of reduced ATP synthase biosynthesis in the cell. The symptoms are severe and often fatal in early newborns with hyperlactacidemia, hypertrophic cardiomyopathy, and high levels of 3-methylglutaconic acid [56,57]. In both types, the ATP production is hampered, leading to energy deprivation. In addition, the hyperpolarization of the mitochondrial membrane, as a result of decreased ATPase activity leads to increased ROS production.

As discussed above, mitochondria are major production sites of superoxide anion, as well as other reactive oxygen species, contributing to cell damage as a consequence of macromolecule oxidation. Furthermore, aberrant ROS production can overwhelm the endogenous antioxidant defense system of the brain, and thus cause further cell death [58]. As several studies have identified ROS as critical players in stroke pathology [58], this presents a potential therapeutic target for ischemic stroke.

Although in ischemic cell mitochondrial dysfunction plays a large role in the generation of ROS, remarkably, oxidative stress is perpetuated via alternative mechanisms as well. NADPH oxidases (NOX) located in microglia, neurons and endothelial cells [59], are paramount in ROS production during ischemic insult. Within the NOX family, NOX2 resides in brain phagocytes and plays a role in the stroke-dependent ROS production [60]. In turn, this contributes to further cell death during ischemia. Therefore, when considering treatment options targeting ROS production, it is essential to acknowledge that ROS are formed by the mechanism discussed above in addition to the mitochondrial pathway at the center of this review. As such, the putative therapeutic effects of NOX inhibition on ischemic stroke have been investigated [60].

The intricate and elaborate mechanism of apoptosis consists of a number of events encompassing mitochondria [61] i.e., release of cytochrome *c*, modifications in electron transport, loss of mitochondrial membrane potential, altered cellular redox state, as well as the influence of pro- and anti-apoptotic Bcl-2 proteins. Members of this family regulate the release of mitochondrial molecules that, once in the cytosol, activate the downstream effectors caspases, a family of conserved cysteine proteases that preside over the controlled demolition and disposal of cellular components [62]. Apoptosis may be triggered by mitochondrial dysfunction via intrinsic and extrinsic pathways [63]. The intrinsic pathway involves the binding of pro-apoptotic factors to the OMM, damaging the mPTP, which allows the release of cell death molecules, including Smac (second mitochondria-derived activator of caspases), AIF (apoptosis-inducing factor), and cytochrome *c*, from the intermembrane space into the cytosol [64]. In this regard, upon migration to the cytosol, Smac binds to and inhibits the inhibitor-of-apoptosis proteins (IAPs), which normally inhibit pro-caspase activation and caspases activity [64]. Conversely, AIF is characterized by the unique capacity to induce caspase-independent chromatin condensation and large-scale DNA fragmentation upon migration to the nucleus, in response to ischemia [65,66]. The formation of an apoptosome, which converts procaspase-9 to caspase-9, is catalyzed by the association of cytochrome *c* with APAF-1, and the subsequent activation of caspase 3. Activated caspase 3 in turn activates endonucleases and proteases, which induce systematic breakdown of chromosomal DNA. This organized and controlled dismantlement is mediated by the expression of ligands for phagocytic receptors, ensuing in phagocytosis [67,68]. In turn, Fas ligand (FasL) or tumor necrosis factor (TNF)-α modulate the extrinsic pathway upon binding to their respective receptors and facilitating the assembly of the death-induced signaling complex (DISC). The conversion of pro-caspase 8 to caspase 8 by the DISC allows for the execution phase of apoptosis, mirroring the intrinsic pathway [69]. Cytotoxic T-cells can induce perforin-granzyme-dependent initiation of the execution phase that also mimics that of the intrinsic and extrinsic pathways [70].

Apoptosis, as well as necrosis and aponecrosis, can lead to cell death in response to inflammation that proceeds after cell swelling and subsequent lysis [71]. An inflammatory response is a secondary cell death process that is harmful to nearby cells, propagating the initial injury [72]. A compensatory mechanism involves a cell survival signaling, and it is usually maintained by phosphokinases such as Akt, which inactivate pro-apoptotic factors Bcl-2-associated X protein (BAX) and Bcl-2-associated-death promoter (BAD) [73]. The calcium/calmodulin phosphatase calcineurin (CaN) can become activated by the large calcium influx associated with excitotoxicity, resulting in the dephosphorylation and activation of said pro-apoptotic factors [74]. The activation of BAD results in its translocation to the OMM and inhibition of survival proteins B-cell lymphoma 2 (Bcl-2) and B-cell lymphoma-extra large (Bcl-xL), signaling BAX to weaken the mPTP contributing to the formation of the apoptosome by the release of cytochrome c, eventually stimulating cell death [75]. In tandem, CaN can dephosphorylate dynamin-related protein 1 (Drp1), triggering mitochondrial fission by the formation of spirals that cleave the mitochondrion, leading to cell death [76]. Although considered a normal physiological process, mitochondrial fission can also indicate pathological conditions. Indeed, the presence of spherical mitochondrial remnants devoid of cytochrome c implies pathological apoptotic events [77].

## 4. Mitochondria-Based Regenerative Medicine

Mitochondrial dysfunction has been recognized in stroke, neurodegenerative diseases, aging, and other metabolic disorders. Therefore, targeting the mitochondrion could be an invaluable therapeutic modality for numerous disease states. Pharmacologic and non-pharmacologic strategies are discussed in the following sections, noting their advantages and disadvantages in correcting mitochondrial deficits.

### 4.1. SIRT1

Dysfunctional mitochondria are involved in several ROS-mediated signaling pathways, which can be responsible for many disease states [78,79]. As such, these pathways represent potential therapeutic targets for the regulation of ROS production. In this regard, the NAD-dependent deacetylase sirtuin 1 (SIRT1) has been shown to improve mitochondrial function and decrease oxidative stress [80]. Highly regulated by the metabolic conditions of the cell, SIRT1 act as redox state and energy sensor [81], linking transcriptional regulation to bioenergetics. SIRT1 is paramount in the metabolism of nutrients such as lipids and glucose via insulin signaling in skeletal muscle, adipose tissue, and the liver [82,83]. Activation of SIRT1 both shields cells from the detrimental effects of inflammation and oxidative stress, and promotes mitochondrial biogenesis and glucose uptake via transcription co-activator of PPARs and PGC1α [84,85]. Resveratrol possesses free radical scavenging properties, and it has been proven an important activator of SIRT1 [86]. Pre-treatment with resveratrol has been demonstrated to have neuroprotective effects following ischemia via the SIRT 1 uncoupling protein 2 pathway (SIRT1-UCP2) [87]. 

### 4.2. Fission and Fusion Modulators

In recent years, our conceptual view of mitochondria has been greatly altered by the discovery that mitochondria exist not only as solitary entities, but function in concert within an integrated network that is constantly remodeled and reorganized by fusion and fission events.

Perturbations of this fine balance have been implicated in a number of diseases, and consequently, have been undertaken as potential therapeutic target [88]. To this end, drugs that alter mitochondrial fission (e.g., Mdivi-1, Dynasore and P110) [89] and fusion (e.g., Leflunomide) [90] cycles have been found to counteract oxidative stress [89,91], with the spatio-temporal distribution and abundance of mitochondria influencing the cell’s energy budget [92], as evidenced by the rapid emission of ATP into the extracellular space in response to hypoxia reducing ischemic damage [93,94].

### 4.3. Purines

Purines have also been shown to possess neuroprotective properties. Moreover, the excitation of exogenous purinergic receptors can maintain cellular energy levels [95]. Purinergic receptor agonists can mitigate the Ca^2+^ imbalance and the over secretion of glutamate, which represent the hallmarks of early ischemia [96]. Selective purinergic agonists protect against stroke through activation of the P2Y1 receptor, increasing astrocyte mitochondrial metabolism and reduces infarct size and edema formation [97]. Normalized mPTP and reduced apoptosis accompany purinergic treatments in stroke animals [98,99].

### 4.4. Methylene Blue

Methylene blue alters the flow of the electrons through the ETC by acting as an electron carrier between NADH and cytochrome *c*. Interestingly, methylene blue is an approved FDA drug for Alzheimer’s disease and Parkinson’s disorders [100], which may advance its use for stroke patients. Methylene blue reduces electron leakage and increases the ATP production by allowing electrons to bypass complex I and III [101]. By reducing electron leakage, methylene blue decreases the ROS production and oxidative stress, thereby dampening neuronal damage [101]. In experimental stroke, methylene blue has been shown to enhance mitochondrial function in vitro, and to promote the activity of complex IV [102]. The cerebral blood flow and glucose uptake of rats that underwent hypoxic conditions and treatment with methylene blue were maintained compared to that of normoxic animals [103]. Non-invasive magnetic resonance imaging reveals that methylene blue decreases the infarct size that correlates to the attenuation of behavioral deficits in in stroke rats. Overall, the studies support methylene blue as a therapeutic agent for stroke.

### 4.5. SOD Mimetics

The imbalance between ROS production and endogenous antioxidants is an underlying mechanism of cell death during ischemia. The mitochondrial Superoxide dismutase 2 (SOD2, or MnSOD) converts superoxide, an extremely harmful and highly reactive radical, to hydrogen peroxide [104,105]. SOD exerts its detoxifying therapeutic effects by alleviating the damage cause by aberrant ROS accumulation after stroke [106]. Overexpression of both SOD1 (Cu/Zn-SOD, cytosolic) and SOD2 have been shown to reduce stroke-related deficits, while deficiencies in these enzymes have been associated with larger infarct volumes [106,107]. However, the short half-life, the relatively high molecular weight, and the low oral bioavailability are limiting factors for SOD to be used as therapeutic agents. Conversely, many SOD mimetics may address these limitations, due to their higher potency, lower molecular weight, high diffusion rate and permeability, lack of immunogenicity, and resistance to peroxynitrate inactivation [108]. Some SOD mimetics contain manganese, which regulates the redox potentials and activities of these chemicals [109,110]. In a stroke model, Manganese (III) tetrakis(1-methyl-4-pyridyl)porphyrin (MnTm4PyP), acted in a dose dependent manner in reducing cytochrome *c* and superoxide radical, and reducing cleaved caspase-3 formation [111]. In a similar fashion, SOD2 mimetics lower superoxide while preserving intracellular calcium levels [111]. Moreover, the Mn(II) pentaazomacrocyclic mimetic M40403 selectively targets superoxide, but when linked with triphenylphosphonium (TPP), the resulting compound, MitoSOD, displays higher redox capabilities against ROS compared to endogenous SOD [110]. Similar therapeutic effects are observed with manganese (III) tetrakis (4-benzoic acid) porphyrin (MnTBAP), as evidenced by decreased oxidative and nitrosative stress [112]. Despite these overwhelming efficacy readouts with these SOD mimetics, adverse effects such as edema formation and even increased cell death during ischemia have been reported [113]. Moreover, while the bulk of studies characterizing SOD mimetics has focused on ischemic stroke models, their utility in hemorrhagic stroke requires further investigation.

### 4.6. Antioxidants

The onset and progression of a number of degenerative disorders is associated with the generation of excess ROS. Efficient scavenging of ROS requires the action of several non-enzymatic and enzymatic cellular antioxidants. An array of natural and synthetic antioxidants is available at present, and their mechanisms of action have been established. Antioxidants such as coenzyme Q, N-acetylcysteine, and vitamins C and E can counteract the deleterious effects exerted by ROS [114] and improve mitochondrial function. However, few clinical trials have succeeded in providing definitive and convincing results on the efficacy of antioxidants (VitE) in the treatment of cardiovascular diseases [114]. Conversely, due to the key role of mitochondria in energy metabolism, cell signaling, apoptosis, Ca^2+^ homeostasis, and ROS production, mitochondria-based treatments have gained considerable attention in recent years as targets for drug-delivery strategies.

Among the antioxidants that can penetrate the mitochondria MitoQ [115], a derivative of ubiquinone, has been shown to decrease lipid peroxidation in experimental models of cardiac hypertrophy and aging [116,117]. The lipophilic triphenylphosphonium (TPP) cation favors MitoQ accumulation inside the mitochondria several hundred-fold compared the untargeted antioxidant [118]. Clinical trials of MitoQ are underway for patients with PD or liver damage [119,120].

Another notable compound that can enter the mitochondria and accumulate inside the organelle is Tiron, an iron chelator and antioxidant which inhibits the production of oxygen radicals, as evidenced by its protective effects against photoaging in human dermal fibroblast [121,122].

MitoVit E (or [2-(3,4-dihydro-6-hydroxy-2,5,7,8-tetramethyl-2*H*-1-benzopyran-2-yl)ethyl]triphenylphosphonium bromide) has been shown to display higher accumulation with 350-fold higher potency compared to non-targeted antioxidants such as vitamin E (or its water-soluble analog trolox) in reducing oxidative stress [123] in a number of animal and cellular models [114].

MitoPeroxidase (2-[4-(4-triphenylphosphoniobutoxy)phenyl]-1,2-benzisoselenazol)-3(2*H*)-one iodide), a mitochondrially targeted analog of ebselen (glutathione peroxidase analog) has been shown to catalyze the breakdown of H_2_O_2_, inhibiting apoptosis induced by oxidants [124]. In cardiovascular diseases, the mitochondria-targeted GSH-analogs appear beneficial in the restoration of the reduced glutathione (GSH) pool, and in the preservation of the mitochondrial redox buffering system and its signaling capacity [125].

These findings suggest that mitochondria-targeted antioxidants, rather than the classically employed ones, may be the chemicals of choice against oxidative stress [126,127] for disease treatment. The application of antioxidants in stroke received much attention with the introduction of NXY-059, a free-radical trapping agent that showed promising neuroprotective effects in animal models of stroke [128]. Unfortunately, in a clinical trial on 3306 patients with acute ischemic stroke, no difference was found between the NXY-059-treated and the placebo group in the frequency of symptomatic or asymptomatic hemorrhage, as well as mortality [129,130]. Rigorous preclinical investigations may reveal better clinical outcomes for another antioxidant called Stilbazulenyl nitrone (STAZN), which has shown improved brain bioavailability due to its high lipophilic characteristic, and high efficiency in inhibiting lipid peroxidation [131,132].

### 4.7. Exercise and Diet

Many studies have demonstrated that exercise, by promoting mitochondrial biogenesis and boosting OXPHOS capacity, provides many benefits for a range of neurological disorders [133,134]. Exercise may modulate mitochondrial function through the AMPK signaling pathway, which can activate PGC1α *via* phosphorylation of threonine and serine residues [135,136], leading to a significant increase in mitochondrial biogenesis and density, mitochondrial respiration, and antioxidant enzymes [137,138]. In addition, the age-dependent decline in mitochondrial functions could be slowed down with exercise [139].

Calorie restriction (CR) has also been reported to be a beneficial prophylactic measure against metabolic disorders and to increase lifespans [140]. Few studies have suggested that CR decreases ROS level and improves mitochondrial functions in humans [140,141]. While the mechanism of CR is not fully understood, among the different mechanisms involved, Sir2/SIRT1 has been shown to modulate the cell-adaptative transcriptional outputs based on its metabolic status [142]. It has been proposed that CR may promote mitochondria biogenesis via deacetylation of PGC1α by activating SIRT1 in response to increase level of NAD+ in tissues [143], suggesting that exercise-mediated AMPK/PGC1 is a potent signaling pathway in enhancing mitochondrial functions. 

## 5. Stem Cells as Source of Healthy Mitochondria

Utilizing stem cells to treat mitochondria dysfunction-related disorders has garnered much interest in the stroke field, with recent reports demonstrating the success of transferring healthy mitochondria into ischemic cells. Following experimental focal ischemia, astrocytes are able to transfer healthy mitochondria to neighboring ischemic neurons [144], illustrating how the dynamic cellular processes of mitochondria are not limited to the intracellular compartment, but encompass the intercellular interaction between astrocytes and neurons after stroke. A similar interaction between healthy mitochondria from stem cells and dysfunctional mitochondria from ischemic neurons would be beneficial for stroke therapy. After transplantation, the long-held dogmatic mechanism of stem cells involves the cells’ migration toward injury site, forming connections and generating new neuronal cells [145]. The current paradigm shift advances the potential of replacing unhealthy mitochondria by intercellular mitochondrial transfer between stem cells and ischemic cells [146,147]. With such transfer of healthy mitochondria, restoration of mitochondrial function, as well as rescue of dying cells after stroke may be possible [148,149]. This phenomenal mitochondria transfer may serve as proof-of-concept that other organelles or organelle-bound units, such as small ions, molecules, microvesicles, lysosomes, exosomes, and endosomes [150] from stem cells can be incorporated into ischemic host cells, allowing repair of bioenergetics functions.

While the explicit details as to the transfer process of mitochondria to host cells are still unclear, evidence suggests that the transfer of mitochondrial genes plays a significant role in correcting the pathophysiology of mitochondrial dysfunction [151]. A pioneering study indicates that the transfer of mitochondria from human stem cells to cells with damaged mitochondria restores mitochondrial respiration [152]. Mechanisms involving the operation of actin based tubes, which entails the formation of tunneling nanotubes (TNTs), or the transfer of mitochondrial fragments or DNA (mtDNA) through vesicles have been shown to actively participate in this transportation process, but the passive uptake of mitochondrial fragments appears not to occur [153].

Stem cells have served as mitochondrial donors in many studies thus far [154]. The transfer of mitochondria has been detected from MSCs to human umbilical vein endothelial cells (HUVEC) previously subjected to in vitro ischemic-reperfusion injury [155]. Aerobic respiration is restored in these cells, as opposed to the lack of respiration in cells cultured alone or alongside MSCs containing dysfunctional mitochondria. Furthermore, the production of phosphatidylserine by damaged cells prompts MSCs to generate TNTs, guiding their migration towards the impaired cells [155]. A similar process ensues as MSCs both increase survival and alleviate cellular damage when introduced to cardiomyocytes exposed to oxygen-glucose deprivation (ischemia) and reperfusion [156]. In parallel, mitochondrial transfer from MSCs to lung epithelium reduces cigarette smoke-induced lung damage [157]. Moreover, the protective effect of MSCs on lung disease in vivo may be mediated by an active degradation process of cells when healthy mitochondria are transferred to lung epithelium and endothelium [158]. MSC may engulf and degrade impaired mitochondria, triggering the activation of heme oxigenase-1 (HO-1), thus prompting mitochondrial biogenesis and yielding increased mitochondrial delivery by MSCs to assist damaged cells in overcoming oxidative stress [159].

The hypothesis that cellular stress is necessary to induce organelle transfer is based on the observation that the transfer of mitochondria rarely occurs when mitochondrial function is generally intact [153]. Mitochondrial transfer appears to be a natural response to an “SOS” distress signal, designed to propel tissue repair in vivo, improving function and cellular bioenergetics [160,161]. Indeed, bone marrow-derived stem cells infused into the trachea of mice and treated with lipopolysaccharide (LPS) display robust attachment to epithelial alveoli cells, as visualized by connexins [162]. Following oligomerization, connexins form gap junctions, allowing cells to connect and transfer small cellular components. The connexin-associated formation of nanotubes and vesicles appears to facilitate the mitochondrial transfer between stem cells and alveolar cells, increasing levels of ATP and production of pulmonary surfactant in alveolar cells [162]. Further investigation of this transfer at a molecular level in both in vitro and in vivo models of asthma reveals that Rho GTPase protein Miro1 plays a key role in connecting mitochondria to cytoskeletal motor proteins, as well as regulating the speed of mitochondrial movement. More importantly, MSC overexpression of Miro1 triggers higher levels of mitochondrial transfer to stressed epithelial cells by TNTs, causing a reduction of inflammatory cell infiltration, cellular apoptosis, collagen deposition, and hypersecretion of mucus in lungs [163]. 

Stem cells also display the ability to donate mitochondria to cancer cells. Mitochondrial transfer from bone marrow MSCs to acute myelogenous leukemia (AML) cells in vitro promote both survival and chemo resistance to doxorubicin [164]. In response to mitochondrial transfer, ATP production in defective cells increases by 50%, and ATP content by 4.5 fold [164]. Even so, there are still unknown mechanisms and signaling pathways regarding the mitochondrial transfer process, namely the degree of cellular impairment necessary to initiate a mitochondrial transfer, and the molecular cues cells use to become attracted to stressed cells, which will be key factors in prompting a mitochondrial transfer towards restoration of function instead of directing damaged cells towards apoptosis [153]. 

**Figure 1:**
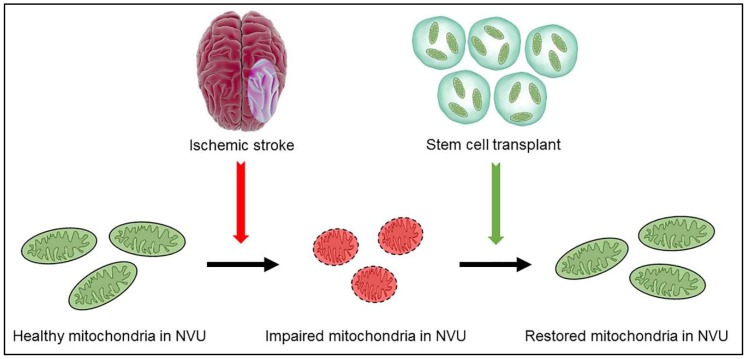
Schematic diagram illustrating mitochondrial replacement strategy. After ischemic stroke, healthy mitochondria in the neurovascular unit (NVU) undergo structure and function impairment. Stem cells transplantation enables the replacement of dysfunctional mitochondria in the NVU with healthy mitochondria from the transplanted stem cells.

The signaling process mitochondria-deficient cells follow when accepting functional mitochondria and its regulation is still uncertain. Evidence suggests, however, that cells have an inherent ability to recognize signs of damage in their stressed counterparts, enabling them to initiate organelle exchange (Figure 1). TNTs are thought to be the most prominent mediators of the inter-cellular mitochondrial exchange process [153]. Their ability to regulate the transfer of small cellular components including vesicles, membrane components, and organelles, has been demonstrated both in vitro and in vivo. TNT formation begins as a membranous protrusion, known as the filopodium, emerges. Upon arriving at the recipient cell, the filopodium is retracted, and releases an ultrafine structure [165]. Mitochondrial exchange may be unidirectional and bidirectional between cells [166,167]. Impeding TNT formation with chemical inhibitors while exposing cells to mechanical stress demonstrates that TNTs are essential components of mitochondrial transfer, and a reduction in transfer efficiency accompanies their inhibition, likely via a receptor-mediated process [168]. Although stress can inhibit the production of TNTs, other stressors can also enhance TNT growth [169], suggesting more in-depth examination into the mechanisms surrounding the specific roles of TNTs in mitochondrial transfer.

Another method of mitochondrial transfer involves extracellular vesicles (EVs) that may act as biomarkers of certain disorders [170,171]. Mitochondrial components have been observed in EVs, but the mechanism underlying this process has yet to be understood. Evidence suggests the influence of EVs in intercellular mitochondrial transfer [152,162], suggesting that the delivery of complete mitochondrial particles through EVs may mediate the reestablishment of mitochondrial function during mitochondrial transfer [153].

Cell fusion provides yet another means for mitochondrial transfer. Human MSCs are shown to fuse to injured or stressed epithelial cells of the respiratory tract [172]. Following myocardial infarction, transplanted bone marrow cells fuse with cardiomyocytes, supporting the idea that stress prompts cellular fusion [173,174]. Improved rodent liver regeneration subsequent to bone marrow transplantation further documents cell fusion [175,176]. Mitochondrial extrusion, allowing the release of mitochondria or its components under specific conditions, may serve as another mechanism of mitochondrial transfer [177,178].

## 6. Stem Cells, Mitochondria, and Stroke

Stem cell therapy to treat ischemic stroke has reached clinical trials, but it remains experimental [179]. That stem cells may transfer viable mitochondria into impaired cells poses as an innovative therapeutic approach for stroke. The use of mitochondrial transfer by stem cells to protect brain tissue from the damage of an ischemic episode appears promising. Mitochondrial transfer from multipotent MSCs to neural cells containing damaged mitochondria reveals that transfer not only restores the bioenergetics of the recipient cells, but also spurs their proliferation [180]. The recognition of Miro1 as a protein requisite to the transfer of mitochondria via TNTs to restore alveolar cells may further enhance the outcome of stem cell-mediated mitochondria transfer. Indeed, Miro1 may play a role in transporting mitochondria from multipotent MSCs to neural cells in experimental stroke [163,180]. MSCs overexpressing Miro1 may contribute to a direct increase in mitochondrial transfer, allowing a greater capacity for mitigating the neurovascular unit deficit consequences of stroke. Additionally, targeting TNTs may facilitate mitochondria transfer from MSCs, as seen with the transfer of fluorescently labeled mitochondria primarily occurring via TNTs [180] (Table 1). In the end, our knowledge of how mitochondria, arguably the powerhouse organelle of the cell, are transferred between cells may pave the way for designing safe and effective mitochondria-based therapies for stroke.

## 7. Conclusions

Novel treatments that target the neurovascular unit in the ischemic brain may prove beneficial in stroke. That the penumbral area in proximity to the core region is characterized by a deficiency in ATP and nutrients during ischemia points to an urgent need to restore mitochondrial function and bioenergetics within this injured region of the brain. Because of the integral role mitochondria play in cell survival, it is critical to target these organelles for stroke therapy. The neurovascular unit in the penumbra region degrades over time in the absence of the appropriate nutrients, making it imperative to find methods of treating this brain tissue in the latter stages of stroke. Recent evidence demonstrating their ability to protect mitochondria in many preclinical trials by way of mitochondria transfer via TNTs, extracellular vesicles, or even cellular fusion, provides compelling evidence to examine the potential of stem cells as a feasible treatment option for stroke. Finding methods designed to transfer healthy mitochondria from stem cells to injured cells stands as a logical approach for treating stroke and other disorders characterized by mitochondrial dysfunction.

## Figures and Tables

**Table 1:** Summary of discussed topics and associated key findings.

Topic	Key Points
Therapeutic options for stroke	Few treatments for stroke exist, which include intravenous thrombolysis and endovascular thrombectomy [1]. Small molecules such as Stachybotrys microspora triprenyl phenol-7 and NSI-189 show promise for treating stroke [3,4].
Mitochondria and stroke	Mitochondria may generate reactive oxygen species that may contribute to diseases such as myocardial infarction and inflammatory conditions [5,9]. Dysfunctional mitochondrial energy generation may lead to Anderson-Fabry disease, which may cause an ischemic stroke [18,19,20].
Mitochondria, ETC, and OXPHOS	The electron transport chain and oxidative phosphorylation processes that occur within the mitochondria are crucial for cellular energy, and thus require optimal function [30,31,32,33]. Defects in the various electron transport chain complex enzymes that facilitate oxidative phosphorylation may lead to different disease pathologies [36,37,38,39,40,41,42,43,44,45,46,47,48,49,50,51,52,53,54,55,56,57]. Altered mitochondrial conditions, such as cytochrome *c* release, electron transport modifications, and changed cellular redox states, may cause downstream pathways to initiate cell death [61,62,63,64,65,66,67,68,69,70,71,72,73,74,75,76,77].
Mitochondria-based regenerative medicine	Mitochondria are a promising therapeutic target for treating stroke, neurodegenerative diseases, aging, and other metabolic disorders. Sirtuin 1, mitochondrial fission and fusion modulators, purinergic agonists, methylene blue, superoxide dismutase mimetics, antioxidants, and proper diet and exercise can improve mitochondrial function and potentially treat diseases associated with mitochondrial dysfunction [78,79,80,81,82,83,84,85,86,87,88,89,90,91,92,93,94,95,96,97,98,99,100,101,102,103,104,105,106,107,108,109,110,111,112,113,114,115,116,117,118,119,120,121,122,123,124,125,126,127,128,129,130,131,132,133,134,135,136,137,138,139,140,141,142,143].
Stem cells as source of healthy mitochondria	Stem cells may be able to transfer healthy mitochondria to ischemic neurons with impaired mitochondria, restoring mitochondrial function in ischemic neurons and rescuing dying neurons after ischemic stroke [146,147,148,149]. Mesenchymal stem cells have successfully transferred healthy mitochondria to various types of impaired cells and repaired cellular damage [155,156,157,158,159]. Mitochondrial transfer may be facilitated by recognition of injured cells and may occur via tunneling nanotubes, extracellular vesicles, or cell fusion [151,152,153,160,161,162,163,164,165,166,167,168,169,170,171,172,173,174,175,176,177,178].
Stem cells, mitochondria, and stroke	Stem cell transfer of viable mitochondria to ischemic cells may be a possible method for treating ischemic stroke. Mitochondrial transfer restores the bioenergetics of the receiving cells and promotes their proliferation [180]. The Miro1 protein facilitates mitochondrial transfer and overexpression of Miro1 may enhance mitochondrial transfer to effectively treat stroke [180].
